# Lifestyle and fertility: the influence of stress and quality of life on female fertility

**DOI:** 10.1186/s12958-018-0434-y

**Published:** 2018-12-02

**Authors:** Stefano Palomba, Jessica Daolio, Sara Romeo, Francesco Antonino Battaglia, Roberto Marci, Giovanni Battista La Sala

**Affiliations:** 1 Unit of Obstetrics and Gynecology, Grande Ospedale Metropolitano “Bianco – Melacrino - Morelli”, Reggio Calabria, Italy; 2 Center of Reproductive Medicine and Surgery, Arcispedale Santa Maria Nuova (ASMN) - Istituto di Ricovero e Cura a Carattere Scientifico (IRCCS), Reggio Emilia, Italy; 3 Department of Morphology, Surgery and Experimental Medicine, University of Ferrara, Ferrara, Italy; 4 Faculty of Medicine, University of Geneva, Geneva, Switzerland; 5 Division of Obstetrics and Gynecology, University Hospital of Geneva, Geneva, Switzerland; 6 University of Modena and Reggio Emilia, Modena, Italy; 7 Center of Reproductive Medicine “P. Bertocchi” Department of Obstetrics and Gynecology, Azienda Unità Sanitaria Locale – IRCCS di Reggio Emilia, Reggio Emilia, Italy

**Keywords:** Infertility, Lifestyle, Quality of life, Sterility, Stress

## Abstract

There is growing evidence that lifestyle choices account for the overall quality of health and life (QoL) reflecting many potential lifestyle risks widely associated with alterations of the reproductive function up to the infertility. This review aims to summarize in a critical fashion the current knowledge about the potential effects of stress and QoL on female reproductive function. A specific literature search up to August 2017 was performed in IBSS, SocINDEX, Institute for Scientific Information, PubMed, Web of Science and Google Scholar. Current review highlights a close relationship in women between stress, QoL and reproductive function, that this association is more likely reported in infertile rather than fertile women, and that a vicious circle makes them to have supported each other. However, a precise cause-effect relationship is still difficult to demonstrate due to conflicting results and the lack of objective measures/instruments of evaluation.

## Background

The original definition of “stress” was about a non-specific body’s response to demand for change and any stimulus able to trigger it was termed as “stressor” [1, 2]. Despite the actual connotation refers to something negative, the concept of stress should be ascribed to the way by which physiological processes and biological tissues are solicited by stressful stimuli. Thus, from a positive point of view, stress can equally represent the ability of a trained body to reach the best athletic performance or the evolutionary pressure at which humans keep on being subjected through ages.

Based on the two dimensions of duration and course, stressors can be distinguished in five categories: 1) *acute time-limited* stressors involving laboratory challenges, such as a public speaking, 2) *brief naturalistic* stressors involving a person confronting a real-life short-term challenge, such as an academic examination, 3) stressful *event sequences*, such as individual events that give rise to a series of related challenges that it is not known when they will subside, 4) *chronic* stressors pervading persons’ life and forcing him/her to restructure social identity and roles, such as suffering a traumatic injury leading to physical disability and 5) *distant* stressors linked to traumatic experiences occurred in the past that yet have the potential to influence people’s life, such as having been sexually assaulted during childhood [3]. This classical classification allowed to clarify how stressful sources may either come from the outside, namely they are generated by the physical environment, job, relationships with others, marital life and all the situations, challenges, difficulties and expectations at which people are faced to daily, or they may be internal factors as well, like the nutritional status, the overall health, fitness levels, and the emotional well-being, that collectively establish the human attitude to respond to, and deal with, external stress-inducing factors.

Unfortunately, there is no consensus in defining and measuring objectively individual body’s stress response but physiological stress can be defined as a wide range of physical responses occurring as a direct effect of a stressor and causing an upset in the homeostasis of the body [4]. The consequence is an immediate disruption of either psychological or physical equilibrium at which the body responds to by stimulating the nervous, endocrine and immune systems and accounting for physical changes with both short- and long-term effects. For example, regular high intensity exercise (i.e. outside stressor) in professional athletes or physically active females may induce menstrual disturbances (i.e. body response to a stressful stimulus or *stress*) due to the endocrine system adaptation to negative energy balance exercise-dependent (i.e. internal stressor) with the following functional/hypothalamic amenorrhea (i.e. altered physical equilibrium). Along the same lines, the individual perception of one’s life in culture and social contexts in which people live (i.e. outside stressor), also called “quality of life” (QoL) [5], constitutes either a positive or negative stressful stimulus of relevance for reproductive purposes (i.e. altered physical equilibrium) and the fertility potential (i.e. body response to a stressful stimulus or *stress* effect) [5–8]. Interestingly, studies in cynomolgus monkeys suggest how the energy imbalance and psychosocial stress might interact synergistically at causing a greater impairment of the reproductive axis than single stressor alone [9].

QoL is a broad ranging concept, incorporating in a complex way individuals’ physical health, psychological state, level of independence, social relationships, personal beliefs and their relationships to salient features of the environment [5]. This definition highlights the view that QoL is subjective, multi-dimensional and includes both positive and negative facets of life [5]. At regard, interesting questions are whether QoL-induced stress contributes to or is a consequence of infertility, and whether a cause-effect relationship can be identified [10–13]. From a different perspective, given that deterioration of QoL or low QoL were associated with infertility and that this latter may account per se for significant levels of mainly psychological stressful stimuli [14, 15], it is remains unclear whether infertility induces negative emotional stress (also called “distress” and opposite to the “eustress”, i.e. positive emotional stress) reflecting in poor QoL or whether a poor QoL accounts for chronic distress during lifespan and finally for infertility.

Based on these considerations, the aim of the present paper will be to comprehensively and critically review the available data regarding the influence of stress and QoL on female reproductive function in order to clarify their relationship(s).

## Methods

We searched all available articles discussing the relationship between stress, QoL and female infertility alone or in concert. Specifically, stress issue was searched throughout its different stressful stimuli and kindred terms including “distress”, “depression”, “anxiety”, “psychological”, “physical”, “physiological” and “emotional stress” as well as issue on QoL was searched using “motherhood”, “sexual attitude”, “marital life”, “life satisfaction” and “work life”. In current analysis, no restriction was used for the different questionnaires to assess the psychological stress and/or the QoL.

Multiple strategies were used to collect relevant demographic, epidemiological, clinical and experimental studies consulting sociological online libraries (IBSS, SocINDEX), Institute for Scientific Information, PubMed, Web of Science and Google Scholar with no language limitations. Studies collected encompass those published up to August 2017. Additional journal articles were included after hand screening of references of collected bibliography.

Since man and women respond to and perceive differently stressful events related to infertility and QoL, specific studies on stress/QoL and male fertility and/or reproductive function in males were excluded from the analysis [14, 16–18]. On the other hand, studies on couples or male population were partially considered not to exclude whether the quality of partnered relationship contributes in defining women’s QoL.

### Stress and infertility

**Fig. 1:**
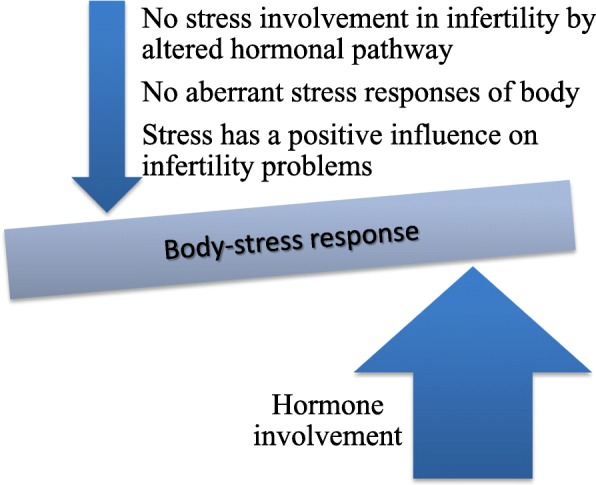
Hormonal involvement in female body-stress response. Since 1967 the majority of studies are in favor of the theory about an aberrant stress response of female body to distress stimuli mediated by hormones changes, whereas only a minority studies provided contradictory statements

The reasonable association between woman’s stress response and fertility potential made literature to accumulate studies with conflicting results [19–29]. However, there is likewise converging evidence on female body-stress response and hormones involvement [30–32] (Fig. 1).

Stressful stimuli cause the activation of the hypothalamic-pituitary-adrenal (HPA) axis and the sympathetic-adrenal-medullary (SAM) axis [33]. The hormones secreted by these systems after stressful stimuli result in an abnormal, prolonged and/or excessive stress-induced body’s set-up that can potentially produce long-term neuroendocrine changes, affecting female fertility [34–39]. Biologically, neurons of the hypothalamic paraventricular nucleus of HPA axis release vasopressin and corticotropin-releasing hormone (CRH) to mediate the secretion of adrenocorticotropic hormone (ACTH) from the anterior lobe of the pituitary gland [33, 40]. In turn, ACTH mediates the secretion of cortisol and glucocorticoid hormones by the adrenal cortex [33, 40].

Differentially, the preganglionic sympathetic fibers of SAM axis, in response to environmental stressful stimuli, activate the adrenal medulla to release epinephrine and norepinephrine into the blood [33]. Experimental data showed that statistically significant reductions in the probability of conception across the fertile window during the first cycle attempting pregnancy were observed for women whose salivary concentrations of α-amylase were in the upper quartiles in comparison with women in the lower quartiles [41]. Even if the salivary α-amylase is considered only a surrogate marker of stress and SAM activity, these data seem to confirm the ability of stress to exert its effect on female fecundity through the SAM pathway [41].

All stress-induced hormones from the adrenal cortex and medulla are responsible for several physiological and mental consequences, which cause the individual to fight with or flight from the stressor. Differences in individual responses could be explained by findings from ewes showing that animals with divergent cortisol responses to ACTH exhibit functional differences in the HPA axis due to innate differences in the gene expression/function of HPA molecules [42]. Further results from female cynomolgus monkeys, exposed to mild combined psychosocial and metabolic stress, show a selected and specific (rather than generalized) increased activity in the adrenal framework significantly related to stress-induced reproductive dysfunction [43].

Increased glucocorticoid release/concentrations leads to profound dysfunction of the hypothalamic-pituitary-ovary (HPO) axis [31, 43–47]. Specifically, distress concentrations of glucocorticoids in the bloodstream reach high levels acting directly on hypothalamus altering the physiologic release of gonadotropin releasing hormone (GnRH) [48, 49]. The synthesis and release of gonadotropins from the pituitary are thus indirectly inhibited, even if a direct pituitary effect of glucocorticoid has been also demonstrated [48, 49]. Accordingly, evidences from animal models are available [41]. In sheep model the infusion of cortisol at concentrations comparable to those produced in humans under stress generates a delay in follicular maturation and ovulation by attenuating or blocking the expected increase of estrogens and luteinizing hormone (LH) surge [41].

However, the signaling pathway by which this occurs remain unclear and is further complicated by the recent findings of kisspeptin (KISS1) and gonadotropin-inhibitory hormone (GnIH). These two neuropeptides induce opposite effects on hypothalamic GnRH release being sensitive to high levels of glucocorticoids [32]. KISS1 exerts stimulatory effects on GnRH secretion [50]. In mouse model, corticosterone administration reduced hypothalamic expression of KISS1 during the estradiol-induced LH surge and decreased the activation of KISS1 neurons [51]. Differentially, GnIH neurons inhibit the activity mediated by either GnRH and KISS1 molecules [52]. Experimental data in ewes demonstrated a direct relationship between both acute and chronic stress and inhibiting GnIH effects on hypothalamus [53] up to inhibition of LH release from the pituitary [54].

Consequently, the stressful stimuli on the female adrenal and HPO axis impact more than one physiological event of fertility including ovulation, fertilization and the implantation rate [34, 48], independently of stimulus origin. Anomalies in the LH pulses induce and inhibition of the ovulatory function directly or thought an effect on sex steroid synthesis/secretion in the ovary [45, 55]. This circumstance can be produced by job-induced stress that exerts its effect through increased LH-plasma concentrations in both the follicular and luteal phases of the ovarian cycle [56].

Both in general and infertile population, distress was respectively associated with decreased conception rates and long menstrual cycles (≥35 days) and lower outcomes of reproductive medicine, including oocytes retrieved, fertilization, pregnancy and live birth rates [11, 41, 57–59]. In addition, in infertile women “chronic” lifetime psychosocial stressors were also identified as detriments to ovarian reserve. Specifically, they were predictive of an enhanced likelihood for diminished ovarian reserve [60]. To this regard, a low socioeconomic status aggravated by sources of stress such as undernutrition and financial hardships potentially plays a key role in affecting ovarian reserve [61].

Of note, the distress can act on female fecundity acting on uterine receptivity also independently from ovarian function. Using a mouse implantation model, the distress induced a poorer endometrial receptivity even if the hormone supplementation was administrated [62].

Depression, high active coping, avoidance and expression of emotions may produce the same consequences on female fecundity [58]. Depression is significantly correlated with the alternative manifestation of stress, i.e. anxiety, affecting cortisol release [44] and symptoms are observed in approximately 37% of infertile women [63]. Consistently, both emotions are prevalent in female partners of infertile couples [64] and more common among females suffering from infertility compared to fertile females [65–67]. The role of emotional distress and anxiety is not still understood, but a small body of evidence suggests that the induction of oxidative stress may be the mechanism by which psychosocial stressors affect oocyte quality through impairment of the overall female health [12, 68, 69].

Many women undergoing reproductive medicine report depressive symptoms prior to beginning their treatments, reflecting a prior history of mood/anxiety disorders independent of infertility itself [63]. Of interest, resilience, i.e. psychosocial stress-resistance, in infertile couples acts as a protective factor against infertility-specific distress and impaired QoL [70] probably through its effect on freedom from anxiety [71]. Moreover, data on psychological interventions or counseling interfering with depression and anxiety are reliable to speculate that the less women are physiologically reactive to distressing stimuli the more they potentially become capable of alleviating their negative consequences on reproductive system [38, 44, 72–83]. Nonetheless, albeit these interventions are effective to optimize natural fertility and outcomes of reproductive medicine strong clinical evidences are still lacking [67, 84, 85].

### QoL and infertility

Although a variety of patient self-reported outcome (PRO) measures are available to investigate the intriguing aspects on the relationship between QoL and infertility (Table 1), only the two Fertility Quality of Life (FertiQoL) and Fertility Problem Inventory (FPI) questionnaires are recently acknowledged as the best useful tools to address this issue in interventional studies [86]. Specifically, the FertiQoL questionnaire is the most widely applied tool and it was developed to tackle limitations of the FPI and other questionnaires designed for specific subpopulations and therefore unable to be used as generic measures for female infertility [87, 88]. The FertiQoL items capture the key life domains affected by fertility problems, including the emotional, mind-body (cognitive and physical), relational and social domains together with the individual perception of the treatment environment and tolerability [87, 88].

**Table 1:** Infertility-related questionnaires exploring patients’ self-reported measures. Questionnaires are characterized by different domains and items and the targeted population

Questionnaire	Items and domains	Target population
Infertility Questionnaire	Self-esteem	Infertile patients
	Blame/guilt	
	Sexuality	
Infertility Reaction Scale	Duration of infertility	Infertile couples who enter an ART treatment program
	Degree of social support effect of infertility on sexual relationship	
	Expected likelihood of achieving pregnancy	
	Anticipation of stress during treatment	
	Self-rating scale of emotional reactions to infertility	
Fertility Problem Inventory	Social concern	Patients seeking for infertility treatment
	Sexual concern	
	Relationship concern	
	Need for parenthood	
	Rejection of childfree lifestyle	
SCREENIVF	State of anxiety	Women and men undergoing infertility treatment cycle
	State of depression	
	Helplessness	
	Lack of acceptance	
	Perceived social support	
Fertility Problems Stress Inventory	Depression	Infertile or presumed infertile couples
	Sexual dissatisfaction	
	Self-esteem	
Infertility Feelings Questionnaire	Adults’ cognitive appraisals of infertility	Patients
Daily Record-keeping Sheet	Negative emotional reactions	Women about to begin a trial of ART
	Physical reactions	
Psychologic evaluation test after ART	Emotional reactions	Women submitted to ART
Concerns about reproductive technologies	Medical aspects	Women submitted to ART
Difficulty with infertility and its treatment	The uncertainty and lack of control	Women undergoing evaluation and treatment of fertility problems
	Family and social pressures	
	Impact on self and spouse	
	treatment-induced problems	
	treatment-related procedures	
Polycystic Ovary Syndrome Quality of Life	Emotions	Women with PCOS
	Body hair	
	Weight	
	Infertility	
	Menstrual problems	
Endometriosis Health Profile-30	Pain	Support group of patients
	Control	
	Powerlessness	
	Emotional well-being	
	Social support	
	Self-image	
	Sexual intercourse	
	Work	
	Relationship with children	
	Feelings about the medical profession, treatment, and infertility	
Fertility Quality of Life	Items that assess core and treatment-related quality of life	People with fertility problems
	Items that assess the overall life	
	Items that assess physical health	

Moreover, there is reasonable evidence for adequate linguistic validation of FertiQoL [86] as confirmed by a plethora of data collected from several populations [8, 89–92]. This support that PROs of FertiQoL reliably measures QoL in women facing infertility and prove that infertility significantly reduces female QoL by increasing anxiety and depression levels [6–8, 89–92]. Both conditions belong to the emotional domain independently of the infertility cause and constitute stressful stimuli (namely distress) acting on the HPA and SAM frameworks as previously described.

For women who have ever met the criteria for infertility and perceive a fertility problem, life satisfaction is significantly lower and the association is weaker for employed women. On the contrary, for women with infertility who do not perceive a problem, not being mother is associated with higher life satisfaction [93]. As consequence, if becoming pregnant is a priority that cannot to be voluntary achieved, this denied attempt affects female QoL and identity with long-term effects and significant higher levels of distress compared to voluntary childlessness women [94].

Unsatisfied motherhood may have implications on female QoL for stress related to marital life too, hampering also couple’s attitude towards successful infertility treatments [59]. Consistently, partnered women who give up a strong intention to have children show more depressive symptoms when relinquished fertility intentions occur in the context of declining relationship quality [95, 96] and in the relational domain, female sexual function positively correlates with male partner sexual function [64]. In addition, infertile women are more likely to underestimate the importance of sexual intimacy in marital life [97] and this is consistent with the deleterious effect of the infertility on sexual dysfunction and poor QoL in women [98, 99]. This scenario can constitute a negative event in women’s life with an impact on QoL because it may potentially trigger chronic distress and subsequently reduce the changes of successful infertility treatments [100]. However, this pathway still needs further clarification [101].

QoL can be impaired in case of reproductive illness at which women are faced to during fertile lifespan. For instance, the polycystic ovary syndrome (PCOS) may be a factor favoring the occurrence of mood disorders as there is evidence that infertile women with PCOS experience high psychological distress and difficulties with coping with their condition as well as poor QoL [102–104]. These and other variables including body mass index, woman’s job, menstrual cycle intervals and sexual satisfaction appear to define QoL in women with PCOS [105]. The validated questionnaire for evaluating the impact of PCOS on health-related QoL in affected women revealed that how weight decrease is of relevance for the overall phenotypic spectrum improvement and a relative decrement in psychological distress [106]. Co-morbidities (for example obesity) may impact many patient’ characteristics, such as social and patient perspective reflected in well-being and QoL individual perception [107].

Moreover, QoL argument is of relevance in Eastern [108, 109] and African [110] societies, where social parenthood cognitions as well as community and family pressure consistently interfere with QoL of infertile women due to the cultural importance of bearing children.

### Stress, QoL and assisted reproductive technologies (ARTs)

Although the influence of stress and distress (measured as anxiety and depression) on ART outcomes was appeared somewhat limited up to 2011 [84], four years later the European Society of Human Reproduction and Embryology (ESHRE) acknowledged the clinical weight of stress and QoL in female reproduction and prompted to incorporate psychosocial assistance into clinical practice of reproductive medicine [111]. In fact, each specific step of ART treatment seems to be closely related to increased levels of distress [112, 113].

This picture seems to be gender-related [114]. During an ART cycle, women show lower levels of QoL compared to men and the number of ART failures in becoming pregnant influences more women’ QoL rather than men [92, 114, 115]. Before knowing ART outcome, women undergoing a cognitive coping and relaxation in their first in vitro fertilization (IVF) cycle showed improved QoL as compared with patients undergoing routine care [116]. From a different perspective, many ART women may report depressive symptoms prior to beginning their cycle, which likely reflects the impact of repeated, unsuccessful, less invasive forms of treatment, but may reflect also a prior history of mood/anxiety disorders independent of infertility [117]. Interestingly, lower concentrations of norepinephrine and cortisol in serum and follicular fluid on the oocyte retrieval day were found in women whose treatments were successful suggesting that both stress-induced biomarkers may negatively influence the clinical pregnancy rate in IVF treatment [118]. Similar findings whereby stress levels where measured in terms of circulating prolactin and cortisol levels suggest that infertile women have a different personality profile in terms of more suspicion, guilt and hostility as compared to the fertile controls [119]. To this regard, the infertility status or its awareness could influence the hormonal release of prolactin/cortisol. On the other hand, the psychological stress may affect the outcome of IVF treatment since anxiety levels in patients who do not achieve pregnancy are higher than in those who become pregnant [119]. Furthermore, women with successful treatment have lower concentrations of adrenaline at oocyte retrieval and lower concentrations of adrenaline and noradrenaline at embryo-transfer day, compared with unsuccessful women [58]. That data emphasizes the positive relationship between adrenal stress-related biomarkers concentrations and pregnancy and depression [58].

## Conclusions

In the current review, we discussed and summarized the literature published over the past years until nowadays concerning the relationship between stress, QoL and female fertility. Much of information stems from cross-sectional and interventional studies in which female population is recruited from clinics of reproductive medicine and kindred registries. Considering that 15% of couples are infertile among general population and a million of couples every year looks for time-consuming and expensive fertility treatment [117], the cohort here argued is not representative of the overall female population. This could reasonably explain some conflicting results cited.

**Fig. 2:**
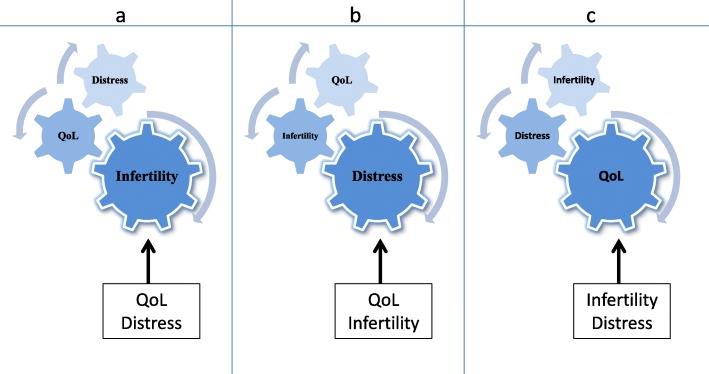
The gearwheel mechanism between infertility, QoL and distress. Depending on which setting **a**, **b** or **c** the mechanism is read into, infertility, distress and QoL can be interchangeably considered the main factor (largest gearwheel) responsible for infertility, QoL and/or distress in females (smallest gearwheels). At their turn, **a**, **b** and **c** mechanisms can be triggered by QoL, distress or infertility (squared boxes), suggesting a mutual and perpetuating effect on female reproductive functions

Mood states are manifestations of well-being encompassing psychological condition and life satisfaction. In this perspective, depression and anxiety represent distress-mediated symptoms of infertility that affects more women than men in four aspects of their life: psychological well-being (depending on the presence or absence of distressing stimuli from any source), marital relationship including sexual intimacy, and QoL. Specifically, most women plan their fertility as meticulously as they do career, educational and lifestyle choices waiting for the right moment of motherhood. In the absence of difficulties, achieving motherhood allows women to reach adult status, social identity, to fulfill gender-role and to complete the marriage. On the other hand, the inability to realize these social expectations can constitute a source of stress and strain resulting in QoL deterioration. This consideration joins others in literature [71, 120, 121] that can be collectively represented by the gearwheel mechanism illustrated in Fig. 2.

On one side, stress from any source has more impact on the wives’ live than husbands’, more impact on satisfaction with self and general well-being than on satisfaction with the marriage or health, and affects QoL mostly indirectly through its impact on the marriage factors. Most results address the alteration of hormonal signaling between the HPA and HPO axis as the more likely mechanism by which stress-related molecules negatively modulate female fertility. Going beyond the emotional fences of depression and anxiety leads women to make the decision to reveal information about their infertility with a resulting positive impact in QoL. Accordingly, literature data show that when a direct disclosure of their infertility issues (i.e. face-to-face, clearly, verbally and with the opportunity for an immediate response) is adopted by women, the perceived support quality from social network members is also related to improved QoL supporting towards infertility treatments [122]. Concisely, when the appropriate infertility patient-centered care is not offered, poor QoL is observed among women [71, 123].

On the other side, QoL and lifestyle choices are non-synonymous concepts, albeit some habits of modern life (classified as social lifestyle factors) can interfere with female health and account for reproductive problems. As consequence, the inability of becoming pregnant can be linked to social behaviors worsening female QoL indirectly.

Thus, it is possible to speculate that information on lifestyle habits should be useful to encourage women by clinicians to improve the overall health because positively affects their ability to reproduce. Moreover, handling the topic of stress with accidently childless couples should be included in routinely cares to minimize the effects of modern life on infertility. In addition, managing the baseline stress (chronic distress) prior to infertility treatment appears to have even greater importance than managing the (acute) stress inherent to fertility treatment itself. This hypothesis is in line with the results of two pilot studies exploring the efficacy of integrative approaches demonstrating that ongoing emotional and instrumental supports are both pivotal to the well-being and QoL of infertile women [82, 124].

This is particularly true for ART population for which health-care providers should be aware of offering psychological support to patients, especially women, during all phases of the medical procedures, given the emotional and physical difficulties associated with this experience. The usefulness of this support has been also acknowledged somewhat of importance to contrast psychological discomfort that could lead to premature termination of ART and consequently to reduce pregnancy rate [13]. For this purpose, it should be also considered that until the desire of motherhood does not become a priority in female life, the presence of an eventual baseline acute and/or chronic stress as low QoL determinants can be not a determinant of such a relevance. However, when the need for ART procedures occurs, it becomes difficult to establish whether ART-stress is related to ART cycle itself (acute or procedural stress, due to the timing and experience during which it arises) rather than QoL-stress, i.e. chronic distress accumulated during lifespan.

**Fig. 3:**
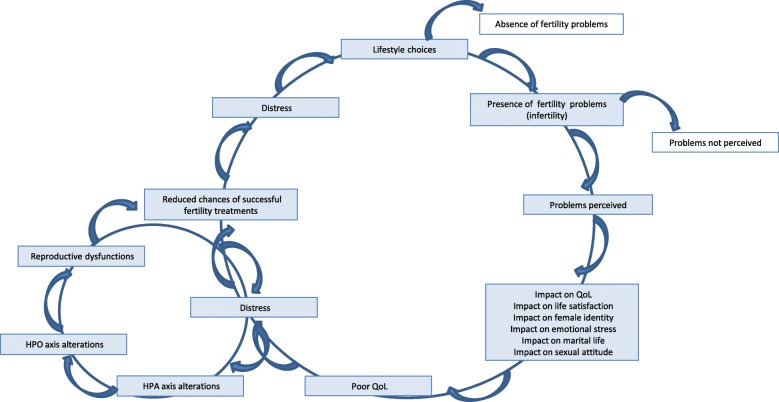
The vicious circle between stress, QoL and altered female fertility. No cause-effect relationship can be assessed inside the intriguing relationship between stress, female infertility and QoL as it mainly depends on what stressor is considered between being infertile and an impaired QoL. Dependently on the individual perception of the problem, infertility can be a serious psychological and relationship stressor that can contribute to poor QoL levels or the clinical consequences of stress from external forms of stressful stimuli. Once infertility is manifested, difficulties arise to establish in which mechanism and because of which reason women become part of the vicious circle

Figure 3 summarizes the theory of vicious circle between stress, QoL and altered female fertility, as suggested by Taymor’s and Bresnick’s hypothesis [125], leaving unresolved the cause-effect question point. However, we can address to further studies the following criticisms of current literature. Determining what is stressful is complex because individual responses to stressful stimuli can differ dramatically converging to the major issue of stress response rather than stress itself. Unfortunately, no optimal stress response marker is available as well as standardized measures defined independently of matching group comparisons. This hampers the possibility to conduct more studies using valid and standard tools as it is actually difficult to reproduce and generalize data from literature in this field. The identification of factors explaining stress, or that may be targets for intervention, would be important to social workers in health care (for instance, to plain programs screening aimed to decrease stress levels). Ultimately, there are quite studies that reported on health-related QoL in infertile couples.

In summary, at the moment the FertiQoL constitutes recommended PROs measures of female infertility related to QoL. Although gaps in evidence remain including test-retest reliability and thresholds for interpreting clinically important changes [84], further use of FertiQoL in future interventional studies is warranted to address the intriguing relationship on the physiological mechanism orchestrating stress and QoL in female fertility.

## Abbreviations

ACTH: Adrenocorticotrophic hormone
ART: Assisted reproductive technology
BMI: Body mass index
CRH: Corticotrophin-releasing hormone
ESHRE: European Society of Human Reproduction and Embryology
FertiQoL: Fertility quality of life
FPI: Fertility problem inventory
GnIH: Gonadotropin-Inhibitory Hormone
GnRH: Gonadotropin releasing hormone
HPA: Hypothalamic-Pituitary-Adrenal axis
HPO: Hypothalamic-Pituitary-Ovary axis
KISS1: Kisspeptin 1
LH: Luteinizing hormone
PCOS: Polycystic ovary syndrome
PRO: Patient self-reported outcome
QoL: Quality of Life
SAM: Sympathetic-Adrenal-Medullary axis
WHO: World Health Organization
